# Solubility Data and Computational Modeling of Baricitinib in Various (DMSO + Water) Mixtures

**DOI:** 10.3390/molecules25092124

**Published:** 2020-05-01

**Authors:** Saad M. Alshahrani, Faiyaz Shakeel

**Affiliations:** 1 Department of Pharmaceutics, College of Pharmacy, Prince Sattam Bin Abdulaziz University, P.O. Box 173, Al-Kharj 11942, Saudi Arabia; sm.alshahrani@psau.edu.sa; 2 Department of Pharmaceutics, College of Pharmacy, King Saud University, P.O. Box 2457, Riyadh 11451, Saudi Arabia

**Keywords:** activity coefficient, baricitinib, co-solvency models, solubility, thermodynamics

## Abstract

The solubility and thermodynamic analysis of baricitinib (BNB) in various dimethyl sulfoxide (DMSO) + water mixtures were performed. The “mole fraction solubilities (*x*_e_)” of BNB in DMSO and water mixtures were determined at “*T* = 298.2–323.2 K” and “*p* = 0.1 MPa” using an isothermal saturation technique. “Hansen solubility parameters (HSPs)” of BNB, pure DMSO, pure water and “DMSO + water” mixtures free of BNB were also estimated. The *x*_e_ data of BNB was regressed well by five different thermodynamics-based co-solvency models, which included “Apelblat, Van’t Hoff, Yalkowsky-Roseman, Jouyban-Acree and Jouyban-Acree-Van’t Hoff models” with overall deviations of <5.0%. The highest and lowest *x*_e_ value of BNB was computed in pure DMSO (1.69 × 10^−1^ at *T* = 323.2 K) and pure water (2.23 × 10^−5^ at *T* = 298.2 K), respectively. The HSP of BNB was found to be closer to that of pure DMSO. Based on activity coefficient data, maximum solute–solvent molecular interactions were observed in BNB-DMSO compared to BNB-water. The results of “apparent thermodynamic analysis” indicated endothermic and entropy-drive dissolution of BNB in all “DMSO + water” combinations including mono-solvents (water and DMSO). “Enthalpy-entropy compensation analysis” showed enthalpy-driven to be the main mechanism of solvation of BNB.

## 1. Introduction

**Figure 1:**
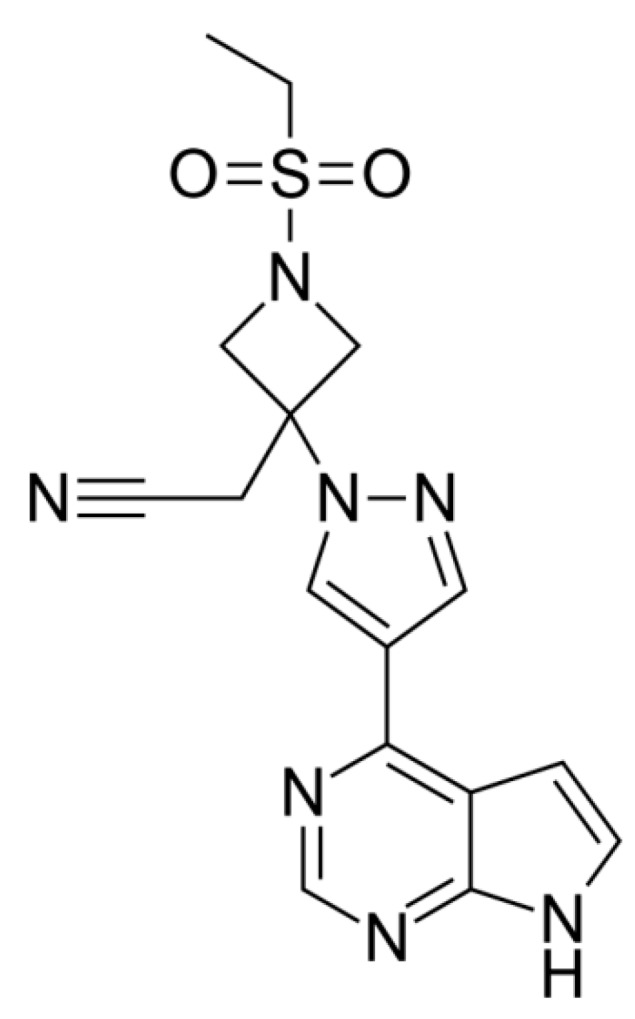
Baricitinib (BNB) chemical structure.

Baricitinib (BNB; Figure 1) is a potent orally active drug that has recently been approved for commercialization by the United States Food and Drug Administration (USFDA) [1,2].

It is a selective irreversible inhibitor of Janus kinase-1 and Janus kinase-2 and shows many therapeutic effects such as anti-inflammatory, immunomodulatory and antineoplastic effects [2,3]. It shows potential results in the treatment of rheumatoid arthritis [3,4]. BNB is reported to be very slightly soluble in water, which creates a lot of problems in its formulation development [2]. BNB shows poor absorption and bioavailability following oral administration in rats [1]. Co-solvency and formulation techniques for solubilization of BNB are poorly reported in the literature. Solubility and thermodynamics data of poorly soluble drugs in neat solvents and water-co-solvent mixtures have greater impact in various fields which includes “medical sciences (preclinical and clinical studies), pharmaceutical sciences (pre-formulation studies and dosage form design), chemical sciences (purification and recrystallization) and physical pharmacy (physicochemical characterization)” [5,6,7,8]. The solubility and solution thermodynamic properties of BNB in some mono-solvents such as water, ethanol, polyethylene glycol-400, ethyl acetate, dichloromethane and dimethyl sulfoxide (DMSO) at “*T* = 298.2–323.2 K” and “*p* = 0.1 MPa” have been reported in literature [8]. The solubilization power of DMSO has been proved in enhancing the solubility of several poorly soluble drugs and bioactive compounds such as sinapic acid, bergenin, naringin, 6-methyl-2-thiouracil and pyridazinone derivative in literature [9,10,11,12,13]. To date, there have been no research reports on the solubility and solution thermodynamic data of BNB in different “DMSO + water” mixtures. Therefore, the current research was aimed at determining the solubility and solution thermodynamic properties of BNB in different “DMSO + water” mixtures at “*T* = 298.2–323.2 K” and “*p* = 0.1 MPa”. The solubility data of BNB obtained in the current research could be useful in “purification, recrystallization, drug discovery and dosage form design” of BNB. Such data could also be beneficial in conducting pharmacokinetic and pharmacodynamics evaluation of BNB in animal models.

## 2. Results and Discussion

### 2.1. Experimental Solubility Data of BNB and Literature Comparison

The “mole fraction solubility (*x*_e_)” values of BNB in mono-solvents (water and DMSO) were computed using Equation (1) and its *x*_e_ values in different “DMSO + water” combinations were calculated by applying Equation (2). The *x*_e_ data of BNB in different “DMSO + water” mixtures including mono-solvents (water and DMSO) at “*T* = 298.2–323.2 K” and “*p* = 0.1 MPa” are tabulated in Table 1.

**Figure 2:**
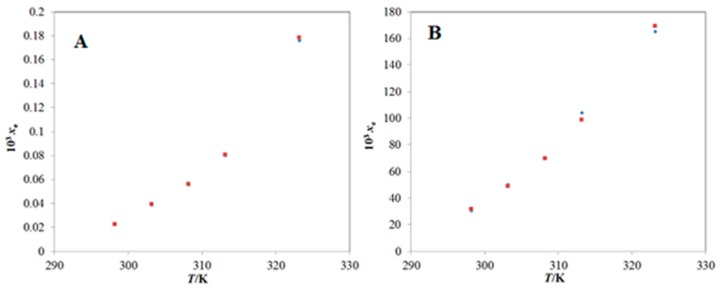
Graphical comparison of solubility of BNB in (**A**) pure water and (**B**) pure dimethyl sulfoxide (DMSO) with literature values at “*T* = 298.2–323.2 K”; the symbol ◼ represents the experimental solubility of BNB in (**A**) pure water and (**B**) pure DMSO and the symbol ◆ represents the literature solubility values of BNB in (**A**) pure water and (**B**) pure DMSO taken from reference [8].

The solubility data of BNB in mono-solvents (water and DMSO) is reported elsewhere [8]. However, there has been no report on the solubility data of BNB in “DMSO + water” mixtures so far. The *x*_e_ value of BNB in pure water at “*T* = 298.2 K” was obtained as 2.25 × 10^−5^ in literature [8]. The *x*_e_ value of BNB in pure water at “*T* = 298.2 K” was computed as 2.23 × 10^−5^ in the current research (Table 1). The *x*_e_ value of BNB in pure DMSO at “*T* = 298.2 K” was obtained as 3.06 × 10^−2^ [8]. The *x*_e_ value of BNB in pure DMSO at “*T* = 298.2 K” was computed as 3.15 × 10^−2^ in the current research (Table 1). The *x*_e_ values of BNB in pure water and pure DMSO computed in the current research were found very close with those reported in literature [8]. The solubility values of BNB in pure water and pure DMSO at “*T* = 298.2–323.2 K” have also been reported [8]. The graphical comparison between *x*_e_ values and literature solubility values of BNB in pure water and pure DMSO at “*T* = 298.2–323.2 K” are shown in Figure 2A,B, respectively.

The results presented in Figure 2A,B indicated good correlation of *x*_e_ values of BNB with its literature solubility values in pure water and pure DMSO at “*T* = 298.2–323.2 K” [8].

**Figure 3:**
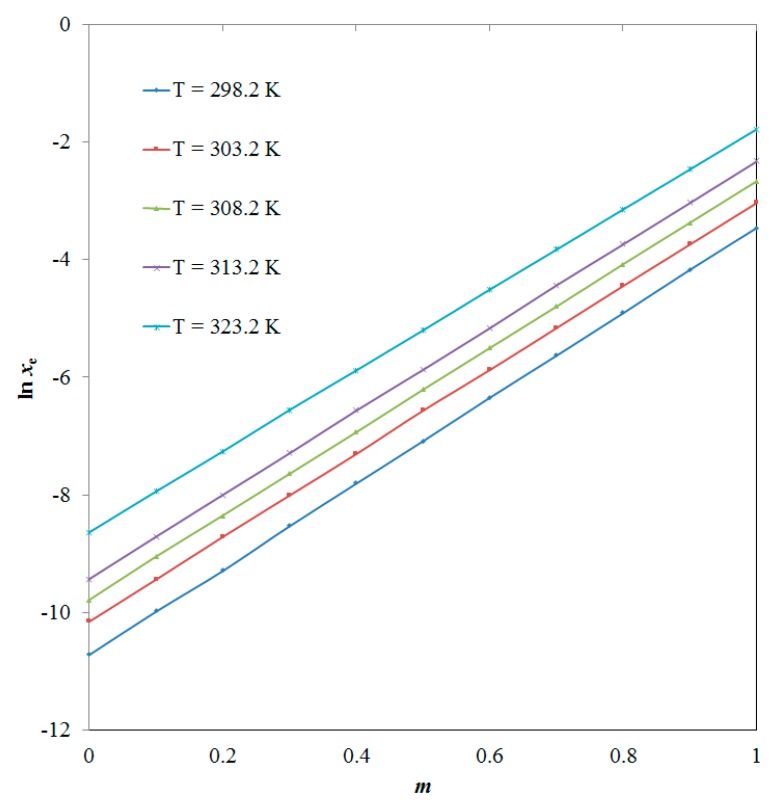
Influence of DMSO mass fraction on solubility of BNB at “*T* = 298.2–323.2 K”; *m* is the DMSO mass fraction in “DMSO + water” mixtures.

According to the results tabulated in Table 1, the *x*_e_ values of BNB were found to increase significantly (*p* < 0.05) with the increase in both DMSO mass fraction (*m*) in “DMSO + water” mixtures and temperature, and hence the lowest *x*_e_ value of BNB was obtained in pure water (*x*_e_ = 2.23 × 10^−5^) at “*T* = 298.2 K” and highest *x*_e_ value of BNB was recorded in pure DMSO (*x*_e_ = 1.69 × 10^−1^) at “*T* = 323.2 K”. The average relative uncertainties in *T*, *m*, *p* and *x*_e_ were recorded as 0.020, 0.001, 0.003 and 0.012, respectively. The highest *x*_e_ value of BNB in pure DMSO was probably due to the lower polarity and HSP of DMSO compared with higher polarity and HSP of water [9,13]. The mass fraction effect of DMSO on BNB solubility at “*T* = 298.15–323.15 K” is shown Figure 3. According to the results summarized in Figure 3, the BNB solubility was observed as increased linearly with increase in DMSO mass fraction at “*T* = 298.15–323.15 K” (*p* < 0.05).

It was also found that the *x*_e_ values of BNB were significantly increased from pure water to pure DMSO and hence DMSO (*p* < 0.05) could be successfully applied as a potential co-solvent in solubilization of BNB.

### 2.2. Hansen Solubility Parameters (HSPs)

The HSPs for BNB and mono-solvents (water and DMSO) were computed by applying Equation (3). However, the HSPs for various “DMSO + water” combinations free of BNB were calculated by applying Equation (4). The results of HSPs are tabulated in Table 2.

The *δ* value of BNB was computed as 28.90 MPa^1/2^, suggesting that BNB had medium polarity. The HSP value for pure DMSO (*δ*_1_) and pure water (*δ*_2_) were found as 23.60 and 47.80 MPa^1/2^, respectively. The *δ*_mix_ values for various “DMSO + water” mixtures free of BNB were found as 26.02–45.38 MPa^1/2^. According to these results, the HSP value of pure DMSO (*δ*_1_ = 23.60 MPa^1/2^) and “DMSO + water” mixtures (at *m* = 0.7−0.9; *δ*_mix_ = 26.02–30.86 MPa^1/2^) were found to close with that of BNB (*δ* = 28.90 MPa^1/2^). The *x*_e_ values of BNB were also found highest in pure DMSO and at *m* = 0.7–0.9 of DMSO in “DMSO + water” combinations. Therefore, the experimental solubility results of BNB in different “DMSO + water” combinations were in accordance with their respective HSPs.

### 2.3. Ideal Solubility and Solute–Solvent Molecular Interactions

The ideal solubility (*x*^idl^) value of BNB was computed using Equation (5) and results are listed in Table 1. The *x*^idl^ values of BNB were computed in the range of 6.84 × 10^−3^–1.54 × 10^−2^ at “*T* = 298.2–323.2 K”. The *x*^idl^ values of BNB were significantly higher than its *x*_e_ values in pure water (*p* < 0.05). However, the *x*^idl^ values of BNB were significantly lower than its *x*_e_ values in pure DMSO at each temperature evaluated (*p* < 0.05). Due to higher solubility of BBN in DMSO, it can also be used as an ideal co-solvent for solubilization of BNB.

The activity coefficient (*γ*_i_) values for BNB in different “DMSO + water” combinations at “*T* = 298.2–323.2 K” were computed using Equation (6) and the results are summarized in Table 3.

The *γ*_i_ value of BNB was found to be highest in pure water at each temperature evaluated. Meanwhile, the *γ*_i_ of BNB was lowest in pure DMSO at each temperature evaluated. The *γ*_i_ value of BNB in pure DMSO (*m* = 1.0) and some co-solvent mixtures (*m* = 0.8 and 0.9) was less than unity. The *γ*_i_ values for BNB were found to decrease significantly from pure water to pure DMSO (*p* < 0.05). The *γ*_i_ values depend on the *x*^idl^ and *x*_e_ values of the drug. In water-rich mixtures, the *x*_e_ values of BNB were much lower than its *x*^idl^ values and hence *γ*_i_ values were high in these mixtures. However, in DMSO-rich mixtures, the *x*_e_ values of BNB were much higher than its *x*^idl^ values, and hence *γ*_i_ values of these mixtures were very low. The highest *γ*_i_ for BNB in pure water could be due to the lowest solubility of BNB in pure water. Based on these results, the highest solute–solvent interactions were found in BNB-DMSO compared with BNB-water.

### 2.4. Thermodynamic Parameters and Dissolution of BNB

The thermodynamic parameters for BNB dissolution in various “DMSO + water” mixtures including mono-solvents (water and DMSO) were computed by “van’t Hoff and Gibbs Equations” (7)–(10) and results are tabulated in Table 4.

The “apparent standard enthalpy (Δ_sol_*H*^0^)” values for BNB dissolution in different “DMSO + water” mixtures including mono-solvents (water and DMSO) were computed as 53.53–64.85 kJ/mol, showing an “endothermic dissolution” of BNB in all “DMSO + water” combinations including mono-solvents (water and DMSO) [13,14]. The “Δ_sol_*H*^0^ values” for BNB dissolution were found to decrease with increase in DMSO mass fraction in “DMSO + water” mixtures and BNB solubility values. Hence, the maximum “Δ_sol_*H*^0^ value” was observed in pure water (64.85 kJ/mol), while the minimum one was computed in pure DMSO (55.53 kJ/mol).

The “apparent standard Gibbs free energy (Δ_sol_*G*^0^)” values for BNB dissolution in different “DMSO + water” combination including mono-solvents (water and DMSO) were computed as 6.80–25.03 kJ/mol (Table 4). The “Δ_sol_*G*^0^ values” for BNB dissolution were also found to decrease with increase in DMSO mass fraction in “DMSO + water” mixtures and BNB solubility values. The maximum and minimum “Δ_sol_*G*^0^ values” for BNB dissolution were found in pure water (25.03 kJ/mol) and pure DMSO (6.80 kJ/mol), respectively. The positive “Δ_sol_*G*^0^ and Δ_sol_*H*^0^ values” suggested an “endothermic dissolution” of BNB in all “DMSO + water” combinations including mono-solvents (water and DMSO) [13].

The “apparent standard entropy (Δ_sol_*S*^0^)” values for BNB dissolution in different “DMSO + water” combinations including mono-solvents (water and DMSO) were computed as 128.89-151.23 J/mol/K, showing an “entropy-driven dissolution” of BNB in all “DMSO + water” combinations including mono-solvents (water and DMSO) [14]. The relative uncertainties in “Δ_sol_*H*^0^, Δ_sol_*G*^0^ and Δ_sol_*S*^0^” were computed as 0.06, 0.38 and 0.05, respectively. Based on these results, the overall BNB dissolution has been proposed as an “endothermic and entropy-driven” in all “DMSO + water” mixtures including mono-solvents (water and DMSO) [13,14].

### 2.5. BNB Solvation Property and Co-Solvent Action

**Figure 4:**
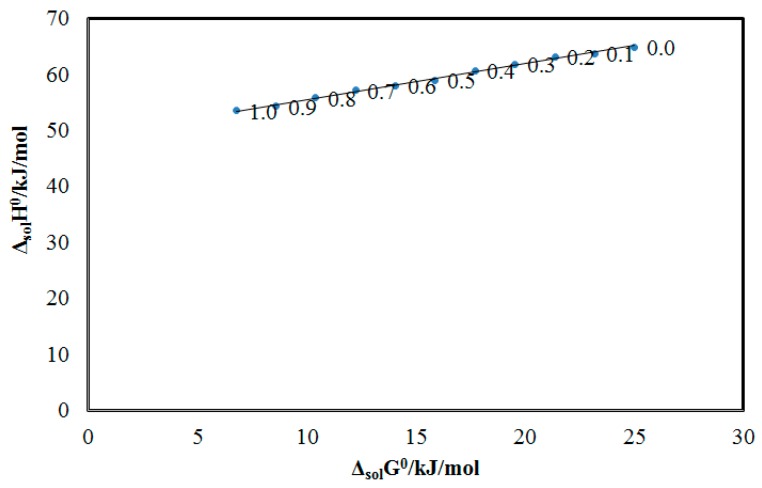
Apparent standard enthalpy (Δ_sol_*H*^0^) vs. apparent standard Gibbs energy (Δ_sol_*G*^0^) enthalpy-entropy compensation graph for solubility of BNB in various “DMSO + water” mixtures at the mean harmonic temperature (*T*_hm_) = 308.96 K.

BNB solvation property and co-solvent action for BNB in different “DMSO + water” combinations including mono-solvents (water and DMSO) were evaluated by applying “enthalpy-entropy compensation analysis” and the resulting data is shown in Figure 4.

It was noticed that BNB in all “DMSO + water” mixtures including mono-solvents (water and DMSO) presented a linear “Δ_sol_*H*^0^ vs. Δ_sol_*G*^0^” relationship with a positive slope value of 0.64. According to these results, the “driving mechanism” for BNB solvation was considered to be “enthalpy-driven” in all “DMSO + water” mixtures including pure water and pure DMSO. These results could be due to the maximum solvation of BNB in neat DMSO molecules in comparison to the molecules of neat water [13]. The BNB solvation property and co-solvent action of DMSO for BNB in different “DMSO + water” mixtures found in the current research was in good agreement with those suggested for the solvation property of sinapic acid, bergenin, naringin, 6-methyl-2-thiouracil and pyridazinone derivative in different “DMSO + water” mixtures [9,10,11,12,13].

### 2.6. Modeling of BNB Solubility

The obtained solubility data of BNB was correlated using five different thermodynamics-based co-solvency models which include “Van’t Hoff, Apelblat, Yalkowsky–Roseman, Jouyban–Acree and Jouyban–Acree–Van’t Hoff models” [15,16,17,18,19,20,21]. Results of “Van’t Hoff model” for BNB in different “DMSO + water” mixtures and mono-solvents (water and DMSO) are tabulated in Table 5.

The “root mean square deviation (*RMSD*)” values for BNB in different “DMSO + water” mixtures including mono-solvents (water and DMSO) were computed as 3.33–5.14% with an overall *RMSD* of 4.09%. Moreover, the “determination coefficient (*R*^2^)” values were computed as 0.9942–0.9972.

Results of “Apelblat model” for BNB in various “DMSO + water” combinations including mono-solvents (water and DMSO) are tabulated in Table 6.

**Figure 5:**
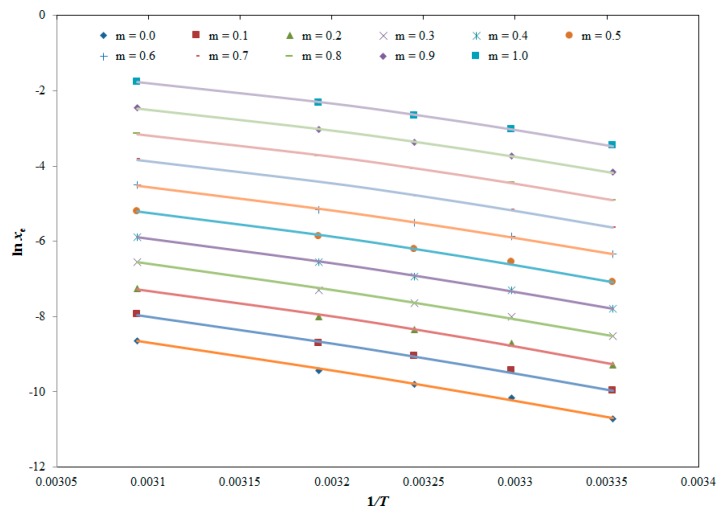
Graphical correlation of solubility of BNB with “Apelblat model” in various “DMSO + water” mixtures at “*T* = 298.2–323.2 K” (Apelblat solubility of BNB is indicated by solid lines and experimental solubility of BNB is shown by the symbols).

The graphical fitting between *x*_e_ and “Apelblat model solubility (*x*^Apl^)” values of BNB are shown in Figure 5 which presented good graphical fitting between *x*_e_ and *x*^Apl^. The *RMSD* values for BNB in different “DMSO + water” mixtures including mono-solvents (water and DMSO) were computed as 1.44–4.16% with an overall *RMSD* of 2.63%. Moreover, the *R*^2^ values were computed as 0.9965–0.9999.

Results of “Yalkowsky-Roseman model” for BNB in different “DMSO + water” mixtures are tabulated in Table 7.

The *RMSD* values for BNB in different “DMSO + water” combinations were computed as 0.87–1.56% with an overall *RMSD* of 1.26%.

Results of “Jouyban–Acree model” for BNB in “DMSO + water” combinations are tabulated in Table 8. The overall *RMSD* value for BNB was computed as 0.98%.

Results of “Jouyban–Acree–Van’t Hoff model” for BNB in “DMSO + water” combinations are also summarized in Table 8. The overall *RMSD* value for BNB was computed as 0.76%.

According to the results obtained for computational modeling, all five co-solvency models expressed low *RMSD* values (overall *RMSD* < 5.0%), which expressed good regression of experimental solubility values of BNB with all co-solvency models evaluated. Nevertheless, it should be pointed out that the error values of each co-solvency model could not be comparable to each other. The “Yalkowsky–Roseman model” is known to correlate the solubility values at various co-solvent mixtures at the given set of temperatures. However, the “Van’t Hoff and Apelblat models” are known to correlate the solubility data of solute at different temperatures in the given set of co-solvent mixtures. On the other hand, the “Jouyban–Acree and Jouyban–Acree–Van’t Hoff models” correlate the solubility data of solute at various temperature with different co-solvent mixtures. Based on the recorded results, all five co-solvency models performed well but “Jouyban–Acree model” has been proposed as the most precise and accurate for this purpose because it uses the least number of model parameters.

## 3. Conclusions

This study aimed to compute the solubility of BNB in various “DMSO + water” mixtures including mono-solvents (water and DMSO) at “*T* = 298.2–323.2 K” and “*p* = 0.1 MPa”. BNB solubility was found to be enhanced with an increase in both DMSO mass fraction and temperature in all “DMSO + water” combinations including mono-solvents (water and DMSO). Measured solubility values of BNB regressed well with five different co-solvency models which includes “Apelblat, Van’t Hoff, Yalkowsky–Roseman, Jouyban–Acree and Jouyban–Acree–Van’t Hoff” models with an overall *RMSD* of <5.0%. The performance of all the studied models was good based on *RMSD* values. However, based on *RMSD* values and use of least number of model coefficients, the Jouyban–Acree model is proposed as the most precise and accurate. The highest solute–solvent interactions were observed in BNB-DMSO combination in comparison to BNB-water combination. “Apparent thermodynamic analysis” indicated an “endothermic and entropy-driven” dissolution of BNB in different “DMSO + water” combinations including mono-solvents (water and DMSO). “Enthalpy-entropy compensation” analysis showed that the solvation property of BNB was “enthalpy-driven” in all “DMSO + water” mixtures including pure water and pure DMSO.

## 4. Experimental

### 4.1. Materials

BNB and DMSO were obtained from “Beijing Mesochem Technology Co. Pvt. Ltd. (Beijing, China)” and “Sigma Aldrich (St. Louis, MO, USA)”, respectively. Water was obtained from “Milli-Q water purification unit”. The properties of materials are tabulated in Table 9.

### 4.2. BNB Solubility Determination

A reported isothermal saturation shake flask method was used to determine BNB solubility in various “DMSO + water” mixtures including mono-solvents (water and DMSO) [22]. The experiment was carried out at “*T* = 298.2–323.2 K” and “*p* = 0.1 MPa” in triplicates (*n* = 3.0). Excess BNB solid was dispensed into transparent glass vials having 1.0 g of each “DMSO + water” combinations including mono-solvents (water and DMSO). The glass vials were placed on a “OLS 200 Grant Scientific Biological Shaker (Grant Scientific, Cambridge, UK)” after the setting of temperature and speed. After 72 h of equilibrium/saturation time, the saturated solutions were taken from the shaker, centrifuged and diluted with mobile phase and subjected for the determination of BNB concentration using reported “high-performance liquid chromatography” method at 265 nm [23]. BNB concentration in the above saturated solutions was obtained using a previously developed calibration curve. The *x*_e_ value of BNB was computed by applying the following equations [14,15]:

(1) $$x_{e}=\frac{m_{1}/M_{1}}{m_{1}/M_{1}+m_{2}/M_{2}}$$

(2) $$x_{e}=\frac{m_{1}/M_{1}}{m_{1}/M_{1}+m_{2}/M_{2}+m_{3}/M_{3}}$$

Here, *m*_1_ = BNB mass; *m*_2_ = DMSO mass; *m*_3_ = water mass; *M*_1_ = BNB molar mass; *M*_2_ = DMSO molar mass and *M*_3_ = water molar mass.

### 4.3. Computation of HSPs

It has been reported that if the solubility parameter of the drug is similar to those of the pure solvents or co-solvent mixtures, the solubility of drug will reach maximum in those particular pure solvent or co-solvent mixtures [24]. Hence, the HSPs for BNB, mono-solvents (water and DMSO) and different “DMSO + water” combinations free of BNB were calculated to compare experimental solubility data. The HSP value (*δ*) for BNB and mono-solvents (water and DMSO) was computed by applying the following equation [24,25,26]:

(3) $$\delta^{2}=\delta_{d}^{2}+\delta_{p}^{2}+\delta_{h}^{2}$$

Here, “*δ* = total HSP; *δ*_d_ = dispersion HSP; *δ*_p_ = polar HSP and *δ*_h_ = hydrogen-bonded HSP”. The HSP values for BNB and mono-solvents (water and DMSO) were computed by “HSPiP software (version 4.1.07, Louisville, KY, USA)” [24]. Meanwhile, the HSPs of different “DMSO + water” combinations free of BNB (*δ*_mix_) were computed by applying the following equation [15,27]:

(4) $$\delta_{\mathrm{mix}}=\propto \delta_{1}+\left(1-\propto \right)\delta_{2}$$

Here, *α* = volume fraction of DMSO in “DMSO + water” mixtures; *δ*_1_ = HSP of pure DMSO and *δ*_2_ = HSP of pure water.

### 4.4. Ideal Solubility and Activity Coefficients

The *x*^idl^ values of BNB at “*T* = 298.2–323.2 K” were computed using the following equation [28]:

(5) $$\ln\;x^{\mathrm{idl}}=\frac{-\Delta H_{\mathrm{fus}}\left(T_{\mathrm{fus}}-T\right)}{RT_{\mathrm{fus}}T}+\left(\frac{\Delta C_{p}}{R}\right)[\frac{T_{\mathrm{fus}}-T}{T}+\ln\left(\frac{T}{T_{\mathrm{fus}}}\right)]$$

Here, *T* = absolute temperature; *T*_fus_ = BNB fusion temperature; *R* = universal gas constant; ∆*H*_fus_ = BNB fusion enthalpy and ∆*C*_p_ = difference in the molar heat capacity of BNB solid state with that of BNB liquid state [28,29]. The *T*_fus_, ∆*H*_fus_ and ∆*C*_p_ values for BNB were taken as 487.42 K, 41.11 kJ/mol and 84.34 **J**/mol/**K**, respectively from reference [8]. The *x*^idl^ values for BNB were now computed using Equation (5).

The *γ*_i_ values for BNB in different “DMSO + water” mixtures including mono-solvents (water and DMSO) were computed using the following equation [28,30]:

(6) $$\gamma_{i}=\frac{x^{\mathrm{idl}}}{x_{e}}$$

The molecular interactions between solute and the solvents were explained based on BNB *γ*_i_ values.

### 4.5. Thermodynamic Parameters of BNB

The thermodynamic dissolution property of BNB in different “DMSO + water” combinations including mono-solvents (water and DMSO) was studied by applying “apparent thermodynamic analysis”, which is based on “Van’t Hoff and Gibbs Equations” at equilibrium. This analysis was carried out at equilibrium by considering the ideality of solution and hence this analysis is called as “apparent thermodynamic analysis”. The “Van’t Hoff Equation” was applied to determine thermodynamic parameters of BNB in different “DMSO + water” combinations which was applied at “mean harmonic temperature (*T*_hm_)” = 308.96 K at “*T* = 298.2–323.2 K” is expressed by the following equation [28,31]:

(7) (∂ln xe∂(1T−1Thm))P=−ΔsolH0R

By plotting ln *x*_e_ versus 1T−1Thm, the Δ_sol_*H*^0^ and Δ_sol_*G*^0^ values for BNB dissolution were obtained from the slope and intercept, respectively, using the following equations [32]:

(8) ΔsolH0=−R(∂ln xe∂(1T−1Thm))P

(9) $$\Delta_{\mathrm{sol}}G^{0}=-RT_{\mathrm{hm}}\times \mathrm{intercept}$$

The Δ_sol_*S*^0^ values for BNB dissolution in different “DMSO + water” combinations including mono-solvents (water and DMSO) were calculated using the following equation [28,31,32]:

(10) $$\Delta_{\mathrm{sol}}S^{0}=\frac{\Delta_{\mathrm{sol}}H^{0}-\Delta_{\mathrm{sol}}G^{0}}{T_{\mathrm{hm}}}$$

### 4.6. BNB Solvation Property and Co-Solvent Action

BNB solvation property and co-solvent action of DMSO for BNB in different “DMSO + water” combinations including mono-solvents (water and DMSO) was studied by applying an “enthalpy-entropy compensation analysis” [14,31]. Such analysis was carried out by plotting the weighted graphs of “Δ_sol_*H*^0^ vs. Δ_sol_*G*^0^” at *T*_hm_ = 308.96 K [14].

### 4.7. Thermodynamics-Based Computational Models

The obtained solubility data of BNB in various “DMSO + water” combinations including mono-solvents (water and DMSO) was correlated by five different co-solvency models which includes “Van’t Hoff, Apelblat, Yalkowsky–Roseman, Jouyban–Acree and Jouyban–Acree–Van’t Hoff” models [15,16,17,18,19,20,21]. The information regarding model is presented in the following subsections:

#### 4.7.1. Van’t Hoff Model

The *x*^Van’t^ value of BNB in various “DMSO + water” combinations including pure water and pure DMSO can be computed using the following equation [15]:

(11) $$\ln\;x^{{\mathrm{Van}}^{\prime }t}=a+\frac{b}{T}$$

Here, *a* and *b* = model coefficients of Equation (11), which were computed by plotting the graphs between ln *x_e_* of BNB and 1/*T*/K.

The regression between x_e_ and x^Van’t^ values of BNB was performed by RMSD and R^2^. The RMSD values of BNB were computed using its standard equation reported in literature [33].

#### 4.7.2. Apelblat Model

The *x*^Apl^ value of BNB in various “DMSO + water” mixtures including pure water and pure DMSO was computed using the following equation [16,17]:

(12) $$\ln\;x^{\mathrm{Apl}}=A+\frac{B}{T}+C\ln\left(T\right)$$

Here, A, B and C = the model coefficients of Equation (12) which were computed by applying “nonlinear multivariate regression analysis” of x_e_ values of BNB tabulated in Table 1 [15]. The regression between x_e_ and x^Apl^ values of BNB was again carried out using “RMSD and R^2^”.

#### 4.7.3. Yalkowsky–Roseman model

The “logarithmic solubility of Yalkowsky–Roseman model (log *x*^Yal^)” for BNB in different “DMSO + water” combinations was computed using the following equation [18]:

(13) $$\mathrm{Log}x^{\mathrm{Yal}}=m_{1}logx_{1}+m_{2}logx_{2}$$

Here, *x*_1_ = mole fraction solubility of BNB in DMSO; *x*_2_ = mole fraction solubility of BNB in water; *m*_1_ = DMSO mass fraction and *m*_2_ = water mass fraction.

#### 4.7.4. Jouyban–Acree Model

The “Jouyban–Acree model” solubility (*x_m,T_*) of BNB in different “DMSO + water” combinations was computed using the following equation [20,21,34,35,36]:

(14) $$\ln\;x_{m}{,}_{T}=m_{1}\ln x_{1}+m_{2}\;\ln\;x_{2}+\lceil \frac{m_{1\;}m_{2}}{T}\overset{2}{\underset{i=0}{\sum }}\;J_{i}\left(m_{1}-m_{2}\right)\rceil$$

Here, *J*_i_ = model coefficient of Equation (14) and it was computed by applying “no-intercept regression analysis” [37,38]. The regression between *x*_e_ and *x_m,T_* values of BNB was performed in terms of *RMSD*.

#### 4.7.5. Jouyban–Acree–Van’t Hoff Model

The “Jouyban–Acree–Van’t Hoff model” solubility of BNB (*x_m,T_*) in various “DMSO + water” mixtures was computed using the following equation [15,38]:

(15) $$\ln\;x_{m}{,}_{T}=m_{1}\left(A_{1}+\frac{B_{1}}{T}\right)+m_{2}\;\left(A_{2}+\frac{B_{2}}{T}\right)+\left[\frac{m_{1}m_{2}}{T}\;\overset{2}{\underset{i=0}{\sum }}J_{i}\left(m_{1}-m_{2}\right)\right]$$

Here, *A_1_*, *B_1_*, *A_2_*, *B_2_* and *J_i_* = the model coefficient of Equation (15).

### 4.8. Statistical Evaluation

Statistical evaluation was conducted using “Kruskal–Wallis test” followed by Denn’s test using “GraphpadInstat software (San Diego, CA, USA)”. The *p* < 0.05 or equal to 0.05 was taken as significant value.

## Figures and Tables

**Table 1:** Experimental solubilities (*x*_e_) of baricitinib (BNB) in mole fraction in different “dimethyl oxide (DMSO) + water” mixtures at “*T* = 298.2 K–323.2 K” and “*p* = 0.1 MPa” ^a^ (values in parentheses are standard deviations).

*m*	*x*_e_				
	*T* = 298.2 K	*T* = 303.2 K	*T* = 308.2 K	*T* = 313.2 K	*T* = 323.2 K
0.0	2.23 (0.02) × 10^−5^	3.90 (0.03) × 10^−5^	5.61 (0.03) × 10^−5^	8.02 (0.03) × 10^−5^	1.71 (0.01) × 10^−4^
0.1	4.66 (0.04) × 10^−5^	7.99 (0.05) × 10^−5^	1.17 (0.03) × 10^−4^	1.65 (0.03) × 10^−4^	3.57 (0.04) × 10^−4^
0.2	9.29 (0.05) × 10^−5^	1.65 (0.01) × 10^−4^	2.36 (0.02) × 10^−4^	3.36 (0.03) × 10^−4^	7.05 (0.05) × 10^−5^
0.3	1.99 (0.03) × 10^−4^	3.34 (0.04) × 10^−4^	4.79 (0.05) × 10^−4^	6.83 (0.06) × 10^−4^	1.42 (0.06) × 10^−3^
0.4	4.09 (0.01) × 10^−4^	6.77 (0.02) × 10^−4^	9.72 (0.04) × 10^−4^	1.41 (0.03) × 10^−3^	2.79 (0.04) × 10^−3^
0.5	8.43 (0.06) × 10^−4^	1.41 (0.07) × 10^−3^	2.00 (0.08) × 10^−3^	2.83 (0.07) × 10^−3^	5.52 (0.08) × 10^−3^
0.6	1.76 (0.07) × 10^−3^	2.83 (0.07) × 10^−3^	4.07 (0.08) × 10^−3^	5.75 (0.08) × 10^−3^	1.10 (0.09) × 10^−2^
0.7	3.60 (0.06) × 10^−3^	5.78 (0.07) × 10^−3^	8.25 (0.07) × 10^−3^	1.19 (0.08) × 10^−2^	2.18 (0.08) × 10^−2^
0.8	7.42 (0.04) × 10^−3^	1.18 (0.03) × 10^−2^	1.69 (0.05) × 10^−2^	2.40 (0.05) × 10^−2^	4.32 (0.06) × 10^−2^
0.9	1.55 (0.01) × 10^−2^	2.40 (0.03) × 10^−2^	3.44 (0.03) × 10^−2^	4.86 (0.04) × 10^−2^	8.53 (0.04) × 10^−2^
1.0	3.15 (0.02) × 10^−2^	4.86 (0.04) × 10^−2^	6.96 (0.05) × 10^−2^	9.85 (0.05) × 10^−2^	1.69 (0.01) × 10^−1^
*x* ^idl^	6.84 (0.00) × 10^−3^	8.09 (0.01) × 10^−3^	9.56 (0.02) × 10^−3^	1.12 (0.03) × 10^−2^	1.54 (0.04) × 10^−2^

^a^ The mean relative uncertainties (*u*_r_) are *u*_r_(*T*) = 0.020, *u*_r_(*m*) = 0.001, *u*_r_(*p*) = 0.003 and *u*_r_(*x*_e_) = 0.012; *m* is the DMSO mass fraction in “DMSO + water” mixtures.

**Table 2:** Hansen solubility parameters (HSPs; *δ*_mix_/MPa^1/2^) for various “DMSO + water” mixtures free of BNB at “*T* = 298.2 K”.

*m*	*δ*_mix_/MPa^1/2^
0.1	45.38
0.2	42.96
0.3	40.54
0.4	38.12
0.5	35.70
0.6	33.28
0.7	30.86
0.8	28.44
0.9	26.02

*m* is the DMSO mass fraction in “DMSO + water” mixtures.

**Table 3:** Activity coefficients (*γ_i_*) of BNB in different “DMSO + water” combinations at “*T* = 298.2 K–323.2 K” (values in parentheses are standard deviations).

*m*	*γ*_i_				
	*T* = 298.2 K	*T* = 303.2 K	*T* = 308.2 K	*T* = 313.2 K	*T* = 323.2 K
0.0	307.00 (2.64)	208.00 (2.32)	171.00 (1.98)	140.00 (1.54)	87.00 (1.01)
0.1	146.97 (1.87)	101.41 (1.74)	81.60 (1.43)	68.23 (0.88)	43.41 (0.79)
0.2	73.68 (1.00)	49.06 (0.63)	40.59 (0.54)	33.46 (0.44)	21.97 (0.10)
0.3	34.44 (0.37)	24.21 (0.22)	19.94 (0.15)	16.49 (0.12)	10.85 (0.10)
0.4	16.69 (0.13)	11.96 (0.11)	9.83 (0.09)	7.96 (0.09)	5.54 (0.08)
0.5	8.11 (0.07)	5.71 (0.06)	4.76 (0.05)	3.97 (0.04)	2.80 (0.03)
0.6	3.88 (0.05)	2.86 (0.04)	2.34 (0.03)	1.95 (0.01)	1.40 (0.01)
0.7	1.89 (0.01)	1.40 (0.01)	1.15 (0.01)	0.94 (0.01)	0.71 (0.01)
0.8	0.92 (0.01)	0.68 (0.00)	0.56 (0.00)	0.46 (0.00)	0.35 (0.00)
0.9	0.44 (0.00)	0.33 (0.00)	0.27 (0.00)	0.23 (0.00)	0.18 (0.00)
1.0	0.21 (0.00)	0.16 (0.00)	0.13 (0.00)	0.11 (0.00)	0.09 (0.00)

*m* is the DMSO mass fraction in “DMSO + water” mixtures.

**Table 4:** Apparent standard enthalpy (Δ_sol_*H*^0^), apparent standard Gibbs energy (Δ_sol_*G*^0^), apparent standard entropy (Δ_sol_*S*^0^) and *R*^2^ values for BNB dissolution in various “DMSO + water” mixtures ^a^ (values in parentheses are standard deviations).

*m*	Δ_sol_*H*^0^/kJ/Δ	Δ_sol_*G*^0^/kJ/Δ	Δ_sol_*S*^0^/J/Δ/K	*R* ^2^
0.0	64.85 (1.78)	25.03 (0.56)	128.89 (2.85)	0.9963
0.1	63.76 (1.73)	23.18 (0.54)	131.34 (2.92)	0.9966
0.2	63.20 (1.70)	21.37 (0.52)	135.39 (3.11)	0.9944
0.3	61.87 (1.68)	19.53 (0.49)	137.04 (3.25)	0.9971
0.4	60.63 (1.61)	17.71 (0.43)	139.91 (3.31)	0.9973
0.5	58.99 (1.58)	15.88 (0.42)	139.50 (3.35)	0.9954
0.6	58.02 (1.54)	14.06 (0.40)	142.25 (3.41)	0.9973
0.7	57.14 (1.51)	12.24 (0.36)	145.30 (3.48)	0.9959
0.8	55.83 (1.49)	10.42 (0.30)	146.95 (3.51)	0.9951
0.9	54.30 (1.46)	8.60 (0.26)	147.88 (3.55)	0.9956
1.0	53.53 (1.43)	6.80 (0.19)	151.23 (3.62)	0.9945

^a^ Average relative uncertainties are *u*(Δ_sol_*H*^0^) = 0.06, *u*(Δ_sol_*G*^0^) = 0.38 and *u*(Δ_sol_*S*^0^) = 0.05; *m* is the DMSO mass fraction in “DMSO + water” mixtures.

**Table 5:** Results of “Van’t Hoff model” for BNB in different “DMSO + water” mixtures ^a^.

*m*	*a*	*b*	*R* ^2^	*RMSD* (%)	Overall *RMSD* (%)
0.0	15.53	−7808.80	0.9962	4.28	
0.1	15.82	−7677.20	0.9965	4.08	
0.2	16.31	−7610.30	0.9942	5.14	
0.3	16.51	−7450.00	0.9970	3.63	
0.4	16.73	−730.70	0.9972	3.49	
0.5	16.80	−7102.40	0.9952	4.40	4.09
0.6	17.13	−6985.70	0.9972	3.33	
0.7	17.50	−6879.70	0.9957	4.05	
0.8	17.69	−6721.90	0.9950	4.35	
0.9	17.81	−6537.90	0.9955	3.94	
1.0	18.21	−6445.30	0.9943	4.36	

^a^ The average relative uncertainties are *u*(*a*) = 0.05 and *u*(*b*) = 0.30; *m* is the DMSO mass fraction in “DMSO + water” mixtures.

**Table 6:** Results of “Apelblat model” for BNB in different “DMSO + water” mixtures ^a^.

*M*	*A*	*B*	*C*	*R* ^2^	*RMSD* (%)	Overall *RMSD* (%)
0.0	447.54	−27,749.80	−64.09	0.9967	4.04	
0.1	560.700	−32,824.80	−80.83	0.9974	3.54	
0.2	830.49	−45,182.50	−120.79	0.9965	4.16	
0.3	539.47	−31,586.40	−77.58	0.9979	3.17	
0.4	732.59	−40,336.40	−106.20	0.9991	2.31	
0.5	847.06	−45,416.00	−123.17	0.9980	3.11	2.63
0.6	728.54	−39,815.80	−105.54	0.9993	1.95	
0.7	967.49	−50,716.90	−140.94	0.9997	1.92	
0.8	1026.81	−53,286.30	−149.71	0.99796	1.81	
0.9	972.13	−50,574.20	−141.58	0.9999	1.44	
1.0	1076.44	−55,275.50	−157.00	0.9999	1.53	

^a^ The average relative uncertainties are *u*(*A*) = 0.26, *u*(*B*) = 0.21 and *u*(*C*) = 0.27; *m* is the DMSO mass fraction in “DMSO + water” mixtures.

**Table 7:** Results of “Yalkowsky-Roseman model” for BNB in different “DMSO + water” mixtures at “*T* = 298.2–323.2 K”.

*m*	Log *x*^Yal^					*RMSD* (%)	Overall *RMSD* (%)
	*T* = 298.2 K	*T* = 303.2 K	*T* = 308.2 K	*T* = 313.2 K	*T* = 323.2 K		
0.1	−4.33	−4.09	−3.94	−3.78	−3.45	1.47	
0.2	−4.02	−3.78	−3.63	−3.47	−3.15	1.56	
0.3	−3.70	−3.48	−3.32	−3.16	−2.85	1.31	
0.4	−3.39	−3.17	−3.01	−2.86	−2.55	1.25	
0.5	−3.07	−2.86	−2.70	−2.55	−2.26	1.50	1.26
0.6	−2.76	−2.55	−2.39	−2.24	−1.96	1.15	
0.7	−2.44	−2.24	−2.08	−1.93	−1.66	1.23	
0.8	−2.13	−1.93	−1.77	−1.62	−1.36	1.03	
0.9	−1.81	−1.62	−1.46	−1.31	−1.06	0.87	

*m* is the DMSO mass fraction in “DMSO + water” mixtures.

**Table 8:** Results of “Jouyban–Acree” and “Jouyban–Acree–van’t Hoff” models for BNB in “DMSO + water” mixtures.

System	Jouyban-Acree	Jouyban-Acree-van’t Hoff
DMSO + water	*J*_i_ 82,431.00	*A*_1_ 18.21
		*B*_1_ −6445.30
		*A*_2_ 15.53
		*B*_2_ −7808.80
*RMSD* (%)	0.98	*J*_i_ 82,122.00
		0.76

**Table 9:** Materials table used in the experiment.

Material	Δecular Formula	Δar Mass (g/Δe)	CAS Registry No.	Purification Method	Mass Fraction Purity	Analysis Method	Source
BNB	C_16_H_17_N_7_O_2_S	371.41	1187594-09-7	None	>0.99	HPLC	Beijing Mesochem
DMSO	C_2_H_6_OS	78.13	67-68-5	None	>0.99	GC	Sigma Aldrich
Water	H_2_O	18.07	7732-18-5	None	-	-	Milli-Q
